# Oncogene-Induced Senescence Is a Crucial Antitumor Defense Mechanism of Human Endometrial Stromal Cells

**DOI:** 10.3390/ijms241814089

**Published:** 2023-09-14

**Authors:** Artem L. Toropov, Pavel I. Deryabin, Alla N. Shatrova, Aleksandra V. Borodkina

**Affiliations:** 1 Mechanisms of Cellular Senescence Group, Institute of Cytology of the Russian Academy of Sciences, Tikhoretsky Ave. 4, 194064 Saint-Petersburg, Russia; 2 Laboratory of Intracellular Membranes Dynamic, Institute of Cytology of the Russian Academy of Sciences, Tikhoretsky Ave. 4, 194064 Saint-Petersburg, Russia

**Keywords:** endometrial stromal cells, senescence, oncogenes, cancer, HRAS^G12V^

## Abstract

Being the major cellular component of highly dynamic tissue, endometrial stromal cells (EnSCs) are exposed to cycles of proliferation upon hormonal stimulation, which might pose risks for the accumulation of mutations and malignization. However, endometrial stromal tumors are rare and uncommon. The present study uncovered defense mechanisms that might underlie the resistance of EnSCs against oncogenic transformation. All experiments were performed in vitro using the following methods: FACS, WB, RT-PCR, IF, molecular cloning, lentiviral transduction, and CRISPR/Cas9 genome editing. We revealed that the expression of the mutant HRAS^G12V^ leads to EnSC senescence. We experimentally confirmed the inability of HRAS^G12V^-expressing EnSCs to bypass senescence and resume proliferation, even upon estrogen stimulation. At the molecular level, the induction of oncogene-induced senescence (OIS) was accompanied by activation of the MEK/ERK, PI3K/AKT, p53/p21^WAF/CIP^/Rb, and p38/p16^INK4a^/Rb pathways; however, inhibiting either pathway did not prevent cell cycle arrest. PTEN loss was established as an additional feature of HRAS^G12V^-induced senescence in EnSCs. Using CRISPR-Cas9-mediated PTEN knockout, we identified PTEN loss-induced senescence as a reserve molecular mechanism to prevent the transformation of HRAS^G12V^-expressing EnSCs. The present study highlights oncogene-induced senescence as an antitumor defense mechanism of EnSCs controlled by multiple backup molecular pathways.

## 1. Introduction

The endometrium is the inner lining of the uterus, which is essential for human reproduction. During a women’s life, this unique tissue adapts to multiple physiological states, including premenarche, menstrual cycling, pregnancy, and postmenopause [1,2]. Among these states, menstrual cycling is almost continuous during the fertile period, with the exception of pregnancy and lactation. Menstrual cycles are regulated by oscillating levels of estrogen and progesterone, which lead to cyclical growth, differentiation/decidualization, shedding, and subsequent regeneration of two-thirds of the endometrium [1]. At the histological level, the endometrium is composed of a layer of stromal cells invaginated by epithelial glands and covered by the luminal epithelium [2]. The highly dynamic nature of this tissue contributes to the accumulation of somatic mutations in cancer-associated genes, which in turn poses risks for developing cancer in adult women [3,4]. Endometrial cancer is one of the most common gynecologic malignancies worldwide [3]. Interestingly, despite the fact that both epithelial and stromal cells are affected by cyclic hormonal alterations, endometrial cancer is commonly referred to as endometrial carcinoma, while endometrial stromal tumors seem to be rare and unusual types of tumors [5,6]. The latter raises the question regarding the defense mechanisms that might underlie the resistance of endometrial stromal cells (EnSCs) against oncogenic transformation.

Senescence is a well-established tumor-suppressive mechanism [7]. At the cellular level, senescence is considered an important intrinsic stress reaction that prevents the propagation of damaged cells via irreversible cell cycle block [7]. Different types of senescence are commonly distinguished, including replicative and various stress-induced forms [8]. More than 20 years ago, the expression of oncogenes was also shown to trigger senescence [9]. Currently, numerous oncogenes such as HRAS^G12V^, NRAS^Q61R^, BRAFV^600E^ are proven to trigger oncogene-induced senescence (OIS) in various types of cells reviewed in [10]. OIS shares the same basic features as replicative and stress-induced forms of senescence. These features include irreversible proliferation block, enhanced expression of the inhibitors of cyclin-dependent kinases p21^WAF/CIP^ and p16^INK4a^, DNA damage, increased cell size, elevated levels of intracellular reactive oxygen species, and senescence-associated β-galactosidase (SA-β-gal) activity [10]. Plenty of evidence provides strong arguments that OIS serves as the first line of defense against cancer development [11,12,13,14]. Indeed, cells exhibiting features of OIS have been identified in early neoplastic and premalignant lesions in various genetically engineered mouse models, as well as in humans [11,12]. Interestingly, the further progression of a subset of these lesions to more advanced cancer stages is associated with the loss of senescence features [14].

Previously, we have shown that EnSCs are prone to senescence triggered by various stresses, including oxidative stress, heat shock, radiation, and treatment with genotoxic agents [15,16,17,18]. However, the response of EnSCs towards oncogene expression remained uncovered. In the present study, we tested the suggestion that OIS might serve as a defense mechanism for EnSCs against the neoplastic transformation.

## 2. Results

### 2.1. Expression of HRAS^G12V^ Results in a Senescence-Like Phenotype in EnSCs

**Figure 1:**
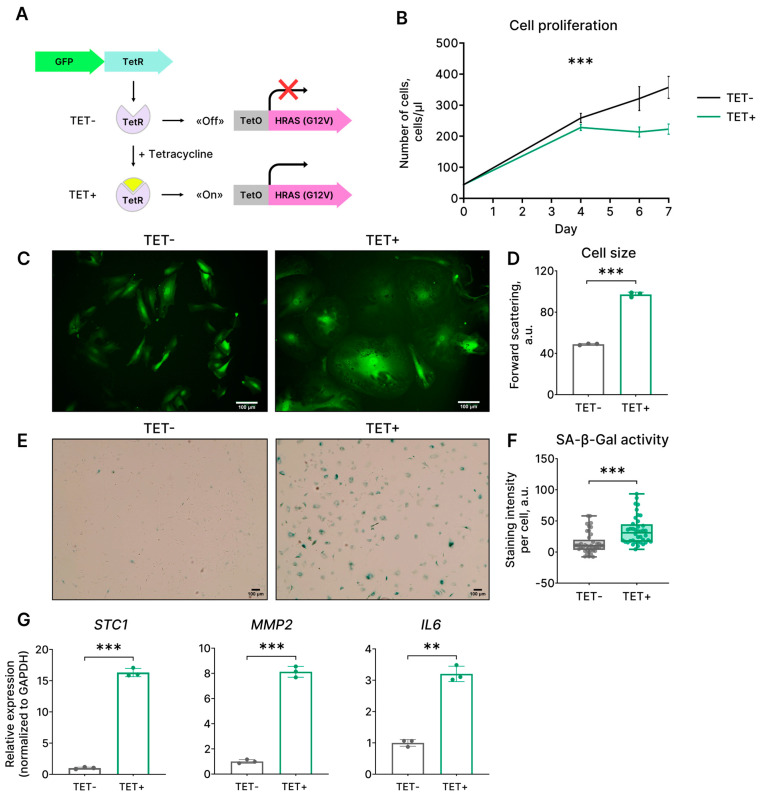
Characteristics of EnSCs expressing mutant oncogene HRAS^G12V^. (**A**) Scheme of the applied two-component Tet-On system for inducible expression of HRAS^G12V^ under tetracycline (TET) stimulation. (**B**) Growth curves, (**C**) morphology, (**D**) cell size, (**E**,**F**) SA-β-Gal activity, and (**G**) mRNA levels of inflammatory factors *STC1*, *MMP2*, *IL6* for the control (TET−) and HRAS^G12V^-expressing (TET+) EnSCs. Scale bars on the microphotographs are 100 μm. (**B**) Data are presented as mean ± SD, *n* = 3, *** *p* < 0.005 by two-way ANOVA (*p*-value corresponds to the interaction “Day”:“Type of cells”). (**D**,**G**) Data are presented as mean ± SD, *n* = 3, ** *p* < 0.01, *** *p* < 0.005 by Welch’s *t*-test. (**F**) Data are presented as median ± 1.5 IQR, *n* = 50, *** *p* < 0.005 by Wilcoxon test. For all experiments, n = 3 means triplicate culture in parallel.

Mutations in the RAS gene occur frequently in various human cancers and have been experimentally validated as the drivers of tumor initiation and maintenance. The missense mutation G12V contributes to HRAS oncogenicity by stabilizing this protein in a constitutively active GTP-bound state [3,4]. To explore the outcomes of HRAS^G12V^ expression in EnSCs, we utilized the tetracycline-controlled system (Tet-On), which allows the inducible expression of the mutant oncogene HRAS^G12V^. The system included two lentivectors. (1) The first one contained the sequence of the mutant oncogene HRAS^G12V^, preceded by the regulatory Tet-operator (TetO) sequence; (2) the second lentivector contained the coding sequences of the repressor protein TetR and of the fluorescent reporter GFP (Figure 1A). In the absence of tetracycline, TetR binds to the TetO sequence and prevents the expression of HRAS^G12V^. Upon addition, tetracycline interacts with TetR, which allows the unhindered expression of the oncogene. Using this system, we initially evaluated the proliferation kinetics in HRAS^G12V^-expressing EnSCs. As shown in Figure 1B, upon tetracycline treatment, EnSCs transduced with the lentivectors for HRAS^G12V^ expression demonstrated a gradual decrease in proliferation rate until its complete loss on day 4 (Figure 1B). Control EnSCs, which carried the same Tet-On lentiviral system but were not treated with tetracycline, preserved normal proliferation rates during the whole observation period (Figure 1B). To validate the acquisition of the senescent phenotype by EnSCs expressing HRAS^G12V^, we further investigated other hallmarks of senescence. As shown in microphotographs, EnSCs expressing HRAS^G12V^ became flattened and vacuolated (Figure 1C). Moreover, upon the expression of the oncogene, EnSCs gradually increased in size (Figure 1D). Next, we revealed SA-β-gal activity in HRAS^G12V^-expressing EnSCs (Figure 1E,F). Finally, we observed enhanced expression of the crucial components of the senescence-associated secretory phenotype (SASP), including *IL6*, *MMP2*, and *STC1*, in EnSCs upon HRAS^G12V^ expression (Figure 1G). Together, the set of identified parameters provides clear evidence in favor of senescence induction in EnSCs expressing HRAS^G12V^.

### 2.2. EnSCs Expressing HRAS^G12V^ Are Unable to Bypass Senescence

**Figure 2:**
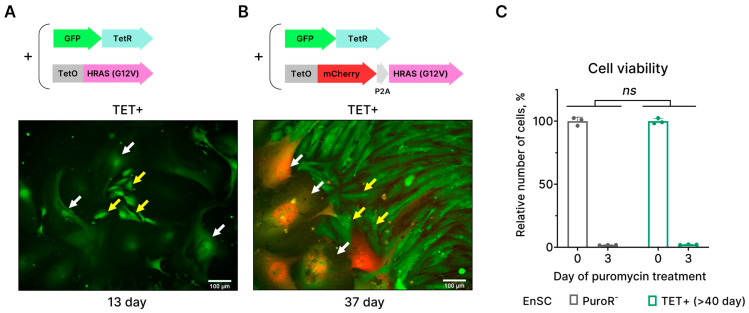
Identification of colonies of small proliferating cells that appear during long-term culturing of HRAS^G12V^-expressing EnSCs as an artifact of imperfect puromycin selection. (**A**,**B**) Schemes of the original and modified two-component Tet-On systems for inducible expression of HRAS^G12V^ under tetracycline (TET) stimulation. Modification resulted in the expression of the fluorescent protein mCherry under the same promoter as HRAS^G12V^. Microphotographs show the absence of mCherry fluorescence in colonies of small cells (indicated with yellow arrows) that appear during long-term culturing of HRAS^G12V^-expressing EnSCs (indicated with white arrows). Scale bars on the microphotographs are 100 μm. (**C**) Relative viability of long-term clonal culture of HRAS^G12V^-expressing (TET+) EnSCs and control non-transduced EnSC under puromycin selection. Data are presented as mean ± SD, *n* = 3, ns *p* > 0.05 by two-way ANOVA (*p*-value corresponds to the interaction “Day”:“Type of cells”). N = 3 means triplicate culture in parallel.

The above results indicate that EnSCs enter senescence upon activation of the oncogene HRAS^G12V^. Although senescence is believed to be an irreversible cell state, there is also evidence indicating that some cells may overcome OIS [14,19]. It has been shown that cells that remained in the senescent state for prolonged periods may resume proliferation and develop features of cancer cells [14,19]. In accordance with this notion, we observed the emergence of small clones of proliferating cells among the senescent ones on day 13 of tetracycline treatment (Figure 2A). Moreover, around day 40 of tetracycline treatment, small proliferating cells almost completely replaced senescent ones. To verify if these cells indeed escaped from HRAS^G12V^-induced senescence, we modified our lentivectors and performed additional experiments. The original lentivector containing the HRAS^G12V^ coding sequence also included the puromycin resistance gene sequence. The latter allowed the selection of HRAS^G12V^-carrying cells on puromycin-containing media; however, this selection may be imperfect. To further control the selection procedure, we cloned the mCherry coding sequence in the same reading frame just before the HRAS^G12V^ sequence. This ensured that both genes were under the same promoter (Figure 2B). Using our modified HRAS^G12V^-mCherry Tet-On system, we visualized HRAS^G12V^-expressing EnSCs by mCherry fluorescence. As expected, at day 37 of tetracycline treatment, we observed large and flattened senescent EnSCs, which were mCherry-positive, and colonies of small cells, which did not express mCherry and thus did not carry HRAS^G12V^ (Figure 2B). This result favors the notion that mCherry-negative small cells might remain after puromycin selection. Indeed, an additional round of puromycin treatment led to the significant death of these small cells, which was comparable to that of the control (non-transduced) EnSCs (Figure 2C). Together, these data provide evidence that EnSCs cannot overcome HRAS^G12V^-induced senescence and suggest a tight molecular regulation of OIS stability in EnSCs.

### 2.3. Estrogen Supplementation Does Not Prevent Proliferation Loss in HRAS^G12V^-Expressing EnSCs

**Figure 3:**
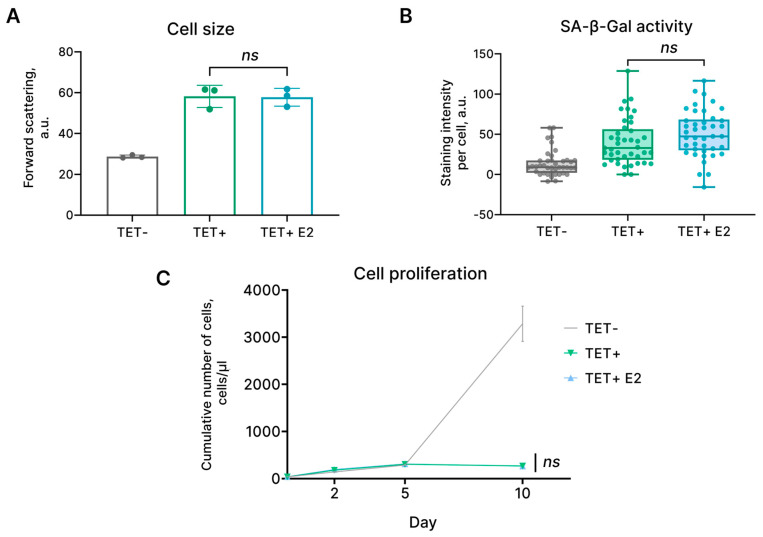
Estrogen supplementation had no effect on the characteristics of HRAS^G12V^-expressing EnSCs. Similar to HRAS^G12V^-expressing cells (TET+), HRAS^G12V^-expressing EnSCs treated with estrogen (TET + E2) were characterized by increased size (**A**), enhanced SA-β-Gal activity (**B**), and proliferation loss (**C**). Untreated control cells are marked as TET−. (**A**) Data are presented as mean ± SD, *n* = 3 (means triplicate culture in parallel), ns *p* ≥ 0.05 by one-way ANOVA with Tukey’s HSD. (**B**) Data are presented as median ± 1.5IQR, *n* = 50; ns *p* ≥ 0.05 by Kruskal–Wallis test with pairwise comparisons by Wilcoxon test and corrections for multiple testing by FDR. (**C**) Data are presented as mean ± SD, *n* = 3 (means triplicate culture in parallel), ns *p* ≥ 0.05 by two-way ANOVA with Tukey’s HSD.

Due to their functional role, EnSCs are exposed to the cyclic influence of estrogen, which stimulates proliferation of these cells crucial for endometrial regrowth during each menstrual cycle [2]. At the same time, there is literary evidence indicating that estrogen might reduce the senescence of various cell types, including endothelial progenitor cells, chondrocytes, and human mesenchymal stem cells [20,21,22]. Therefore, we assessed the effects of estrogen on the main characteristics of HRAS^G12V^-expressing EnSCs. To this end, we supplemented tetracycline-containing growth media with β-Estradiol. Importantly, β-Estradiol had no effect on either proliferation or the size and SA-β-gal activity of the HRAS^G12V^-expressing EnSCs (Figure 3). These results provide additional confirmation of the stability of the senescence reaction of EnSCs upon the oncogene expression.

### 2.4. Molecular Mechanisms Regulating HRASG12V-Induced Senescence in EnSCs

**Figure 4:**
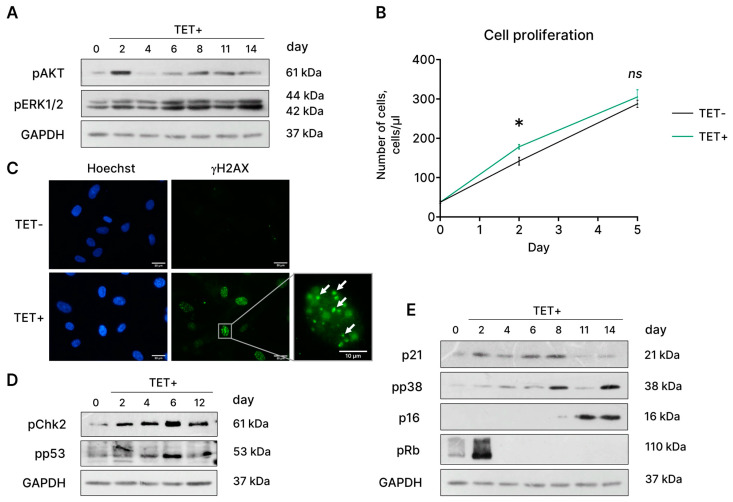
Signaling events mediating hyperproliferation and subsequent proliferation arrest in HRAS^G12V^-expressing EnSCs. (**A**) Increased phosphorylation levels of AKT and ERK1/2 kinases in HRAS^G12V^-expressing (TET+) EnSCs. (**B**) Growth curves reflecting a 5-day period of hyperproliferation of TET+ cells. (**C**) γH2A.X foci reflect DNA damage in TET+ EnSCs. (**D**,**E**) Activation of Chk2/p53/p21^WAF/CIP^/Rb and p38/p16^INK4a^/Rb, two signaling pathways responsible for proliferation arrest in TET+ EnSCs. Untreated control cells are marked as TET−. Scale bars on the microphotographs are 50 μm or 10 μm. For blotting, GAPDH was used as a loading control, and representative blots are shown. Data are presented as mean ± SD; *n* = 3 (means three independent experiments); ns *p* > 0.05, * *p* < 0.05 by two-way ANOVA with Tukey’s HSD.

The main cellular outcomes of RAS activation are proliferation and survival, mediated via the RAS/RAF/MEK/ERK and RAS/PI3K/AKT pathways [23]. In line with that, we observed the phosphorylation of the main downstream targets of RAS—ERK1/2 and AKT—shortly after tetracycline addition (Figure 4A). The latter resulted in a brief period of hyperproliferation of oncogene-expressing EnSCs, as indicated in Figure 4B.

Hyperproliferation caused by oncogenic HRAS^G12V^ drives DNA replication stress and constitutive activation of the DNA damage response (DDR) [24]. Indeed, we detected the accumulation of γH2AX foci, which mark DNA damage, in HRAS^G12V^-expressing EnSCs (Figure 4C). Activation of DDR in EnSCs triggered by the expression of the oncogene led to the phosphorylation of Chk2 and the tumor suppressor protein p53 (Figure 4D). The latter was followed by the enhanced expression of the inhibitor of cyclin-dependent kinases p21^WAF/CIP^ and the establishment of cell cycle arrest (Figure 4E). Another classical molecular pathway responsible for maintaining cell cycle block during senescence is p38/p16^INK4a^/Rb [17]. As depicted in Figure 4E, phosphorylation of the stress kinase p38 increased after 8 days of tetracycline treatment, which further led to the elevated expression of p16^INK4a^. Thus, we detected activation of two major signaling pathways, p53/p21^WAF/CIP^/Rb and p38/p16^INK4a^/Rb, that mediate cell cycle block in HRAS^G12V^-expressing EnSCs. Activation of both pathways was strictly coordinated; expression of p21^WAF/CIP^ started just after tetracycline addition and remained elevated for 8 days, while expression of p16^INK4a^ increased only on day 11 (Figure 4E). The activation dynamics revealed suggest that the p53/p21^WAF/CIP^/Rb pathway is responsible for initiating cell cycle block, whereas the p38/p16^INK4a^/Rb pathway mediates its stabilization during OIS in EnSCs.

**Figure 5:**
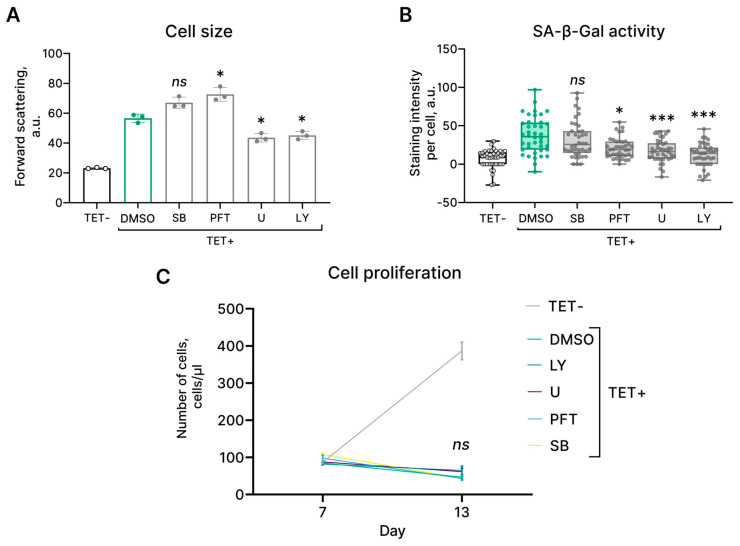
Modulation of activity of the molecular pathways involved in HRAS^G12V^-induced senescence in EnSCs. (**A**) Сell size, (**B**) SA-β-Gal activity, and (**C**) growth curves of the control (TET−), HRAS^G12V^-expressing (TET+) EnSCs, and HRAS^G12V^-expressing EnSCs treated with AKT inhibitor LY294002 (TET+ LY), p53 inhibitor pifithrin-α (TET+ PFT), p38 inhibitor SB203580 (TET+ SB), and MEK1/2 inhibitor U0126 (TET+ U). (**A**) Data are presented as mean ± SD, *n* = 3 (means triplicate culture in parallel), ns *p* ≥ 0.05, * *p* < 0.05 by one-way ANOVA with Tukey’s HSD. (**B**) Data are presented as median ± 1.5IQR, *n* = 50; ns *p* ≥ 0.05, * *p* < 0.05, *** *p* < 0.005 by Kruskal–Wallis test with pairwise comparisons by Wilcoxon test with corrections for multiple testing by FDR. (**C**) Data are presented as mean ± SD, *n* = 3 (means triplicate culture in parallel), ns *p* ≥ 0.05 by two-way ANOVA with Tukey’s HSD.

Having established the crucial molecular pathways that mediate the initiation and progression of HRAS^G12V^-induced senescence in EnSCs, we further tested the possibility of reversing or alleviating this reaction. To this end, we utilized specific inhibitors for each pathway, which included (1) U0126, an inhibitor of the kinase activity of MEK1/2, which prevents the activation of ERK; (2) LY294002, a blocker of PI3K-dependent AKT phosphorylation and kinase activity; (3) SB203580, an inhibitor of the catalytic activity of p38 that binds to its ATP-binding pocket; (4) pifithrin-α, an inhibitor of p53 activity (Figure 5A). The compounds were used at concentrations that were found to be efficient and nontoxic in our previous studies [15,25,26,27]. As shown in Figure 5A,B, U0126 and LY294002 prevented cell size increase and SA-β-Gal staining of HRAS^G12V^-expressing EnSCs, which demonstrates the involvement of both pathways in the acquisition of the senescent phenotype during OIS. At the same time, none of the applied compounds could prevent the loss of proliferation induced by the oncogene expression (Figure 5C). Of note, we previously revealed that cell cycle arrest in stress-induced senescent EnSCs could be overcome by p38 inhibition [15]. These results suggest that cell cycle arrest in HRAS^G12V^-expressing EnSCs is probably controlled by several backup pathways that prevent cell cycle reentry and transformation.

### 2.5. PTEN Loss-Induced Senescence Is a Backup Molecular Mechanism to Prevent the Transformation of HRAS^G12V^-Expressing EnSCs

**Figure 6:**
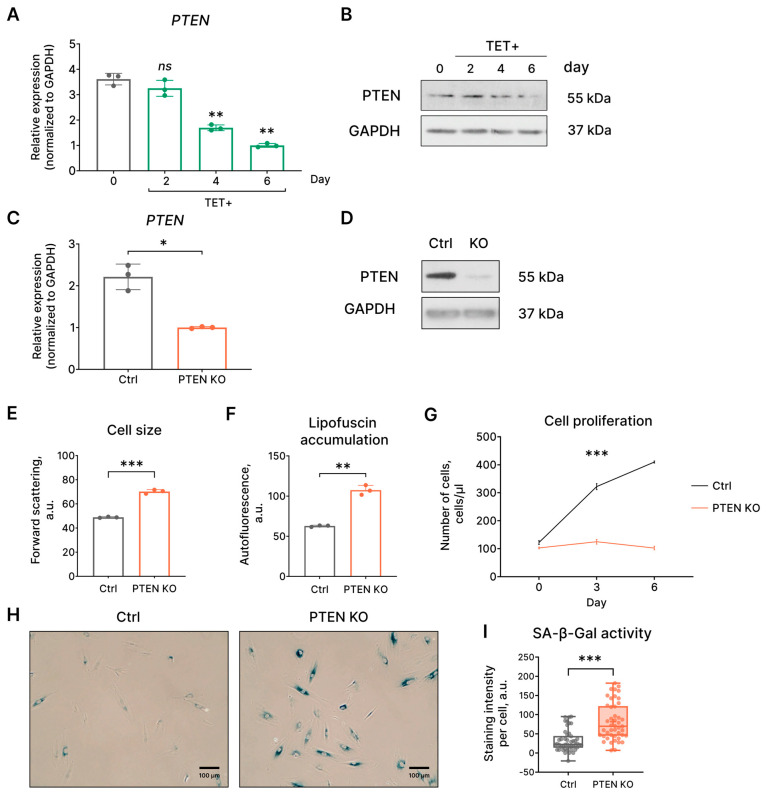
Evidence of PTEN loss-induced senescence of EnSCs. (**A**) mRNA and (**B**) protein levels of PTEN expression in HRAS^G12V^-expressing (TET+) EnSCs. (**C**,**D**) Verification of CRISPR/Cas9-mediated *PTEN* knockout in control EnSCs at mRNA and protein levels. (**E**) Cell size, (**F**) lipofuscin intracellular level, (**G**) growth curves, and (**H**,**I**) SA-β-Gal activity in control and *PTEN* knockout (PTEN KO) EnSCs. Scale bars on the microphotographs are 100 μm. For blotting, GAPDH was used as a loading control, and representative blots are shown. (**A**) Data are presented as mean ± SD, *n* = 3 (means triplicate culture in parallel), ns *p* ≥ 0.05, ** *p* < 0.01 by one-way ANOVA with Tukey HSD. (**C**,**E**,**F**) Data are presented as mean ± SD, *n* = 3 (means triplicate culture in parallel), * *p* < 0.05, ** *p* < 0.01, *** *p* < 0.005 by Welch’s *t*-test. (**G**) Data are presented as mean ± SD, *n* = 3 (means triplicate culture in parallel), *** *p* < 0.005 by two-way ANOVA with Tukey HSD. (**I**) Data are presented as median ± 1.5 IQR, *n* = 50, *** *p* < 0.005 by Wilcoxon test.

Previously, it was shown that the oncogenic HRAS downregulates the expression of the tumor suppressor PTEN, which negatively regulates cell survival by opposing the activation of the PI3K/AKT/mTOR signaling network [23]. The expression of the mutant HRAS^G12V^ in EnSCs resulted in decreased mRNA and protein expression of the crucial tumor suppressor PTEN (Figure 6A,B). As shown in Figure 6A,B, both PTEN mRNA and protein levels gradually declined from day 2 to a minimal level on day 6 of tetracycline treatment. To determine the functional role of PTEN downregulation during the development of HRAS^G12V^-induced senescence of EnSCs, we conducted *PTEN* knockout experiment. As shown in Figure 6C,D, application of the CRISPR/Cas9 genome editing system resulted in a significant reduction in *PTEN* gene expression and almost completely abolished its expression at the protein level. We next assessed the main characteristics of EnSCs with PTEN loss. Notably, EnSCs with *PTEN* knockout demonstrated increased cell size and autofluorescence levels, indicating hypertrophy and lipofucine accumulation, along with complete proliferation loss (Figure 6E–G). Moreover, we detected enhanced SA-β-gal staining of EnSCs with PTEN loss (Figure 6H,I). Together, these data indicate that the loss of PTEN is sufficient to trigger senescence in EnSCs. Thus, the loss PTEN observed in HRAS^G12V^-expressing EnSCs might serve as an additional backup mechanism to induce senescence and thus avoid cell transformation.

## 3. Discussion

The present study uncovered the defense mechanisms of EnSCs against transformation. We revealed that EnSCs are prone to OIS in response to HRAS^G12V^ oncogene expression. The first evidence of HRAS-induced senescence dates back to 1997, when the authors observed stalled mitotic activity and an enlarged and flattened morphology of human diploid fibroblasts expressing mutant HRAS^G12V^ [9]. Later on, this observation was significantly extended, and today, OIS resulting from the expression of different oncogenes is considered an intrinsic antitumor mechanism common to various types of cells [10]. Similar to other cell types, upon HRAS^G12V^ expression, EnSCs acquire all features typical for senescent cells, including proliferation block, altered morphology, and SA-β-Gal activity. Commonly, the loss of proliferation and the development of the senescent phenotype are considered as consequences of DDR resulting from hyper-replication of genomic DNA induced by the oncogene expression [24]. In line with this notion, we observed a brief period of hyperproliferation of HRAS^G12V^-expressing EnSCs followed by proliferation arrest and the emergence of DNA damage.

Despite the concrete mechanism of DNA damage caused by oncogene expression, DDR further leads to cell cycle arrest through the activation of the p53/p21^WAF/CIP^/Rb and/or p38/p16^INK4a^/Rb pathways [17]. Although senescence as the outcome of oncogene expression may appear to be independent of cell type, the molecular mechanisms that establish cell cycle arrest during OIS largely depend on the specific cellular context [28]. For example, the expression of p53 is crucial for the development of HRAS^G12V^-induced senescence in normal human fibroblasts because its depletion prevents proliferation arrest in cells expressing the oncogene [9,24,29]. In contrast, normal human mammary epithelial cells and esophageal keratinocytes undergo HRAS^G12V^-induced senescence in a p53-independent manner [30,31]. The same controversy holds true for the involvement of p16^INK4a^ in HRAS^G12V^-induced senescence. While the progression of OIS in fibroblasts relies on the expression of p16^INK4a^, the depletion of p16^INK4a^ in HRAS^G12V^-expressing normal human melanocytes had no effect on senescence progression [32,33]. Notably, a recent study revealed that the expression of oncogenic HRAS^G12V^, along with p16^INK4a^ knockdown, in EnSCs induced high-grade endometrial stromal sarcoma in mice [34]. Here, we detected activation of both signaling pathways, p53/p21^WAF/CIP^/Rb and p38/p16^INK4a^/Rb, in HRAS^G12V^-expressing EnSCs. However, neither the inhibition of p53 nor the downregulation of p38/p16^INK4a^ affected OIS progression in EnSCs. Moreover, inhibition of ERK and AKT, which are downstream targets of the major signaling pathways directly activated by RAS, reduced the senescent phenotype but also did not affect the cell cycle block in oncogene-expressing EnSCs. These data suggest the existence of compensatory pathways that regulate cell cycle arrest during OIS in EnSCs.

Further analysis of the molecular consequences of HRAS^G12V^ expression in EnSCs revealed the gradual loss of PTEN expression. Earlier, it was shown that oncogenic RAS downregulates the expression of the proapoptotic tumor suppressor PTEN in fibroblasts and epithelial cells through a p53-independent pathway [23]. The authors of the above study experimentally verified that oncogenic RAS may suppress PTEN expression via the RAF/MEK/ERK/c-Jun pathway, leading to cellular transformation. However, in the case of HRAS^G12V^-expressing EnSCs, decreased expression of PTEN did not lead to cellular transformation. By performing an additional set of experiments, we uncovered that the loss of PTEN alone is sufficient to trigger senescence in EnSCs. This result is consistent with the existing literature, which reveals the tumor suppressor loss-induced form of senescence induced by the reduced expression of the *PTEN* gene [35,36]. Furthermore, a recent study uncovered the crucial role of the interplay between p53 and PTEN in cell fate decision, including cell cycle arrest, senescence, autophagy, and apoptosis [37]. It should be highlighted that previous studies considered both HRAS^G12V^- and PTEN-loss-induced forms of senescence separately. However, the present study provides the first evidence of the possible intersection between these forms of senescence. Together, the obtained results allow for the speculation that PTEN loss might serve as a backup mechanism that additionally controls proliferation arrest during HRAS^G12V^-induced senescence of EnSCs.

Despite the tight molecular control of proliferation arrest during OIS, a growing body of evidence demonstrates that cells might escape from OIS through cell-autonomous and cell-non-autonomous mechanisms, including derepression of the hTERT locus, reorganization of topologically associated domains, stemness-associated reprogramming, and downregulation of histone demethylases [14,19]. For example, a previous study revealed that the population of human diploid fibroblasts could spontaneously escape from OIS induced by HRAS^G12V^ [19]. The authors demonstrated preserved proliferation and the decreased expression of p16^INK4a^ in OIS-escaped fibroblasts. Similar to these findings, we detected the emergence of small proliferating EnSCs, which eventually replaced senescent EnSCs from the population. However, we experimentally verified that these small cells originated from imperfect lentiviral transduction and subsequent antibiotic selection. Probably, the results of the above study might also be influenced by the technical batches of the transduction procedure, as OIS-escaped fibroblasts were susceptible to stress-induced senescence and unable to grow in soft agar [19]. According to our data, EnSCs that expressed HRAS^G12V^ remained senescent for prolonged periods and could not overcome OIS and resume proliferation.

Another possibility to escape from OIS that should be taken into account might result from the physiological estrogen-mediated regulation of EnSC proliferation within the endometrium. This possibility is reinforced by the fact that estrogen controls telomerase activity and hTERT expression in various estrogen-targeted tissues, including the endometrium [38]. Indeed, estrogen deficiency leads to telomere shortening, whereas estrogen hyperstimulation increases the telomerase activity and maintains telomere length [39]. Moreover, estrogen supplementation has been found to be effective in reducing the senescence of various types of cells [20,21,22]. Notably, HRAS^G12V^-expressing EnSCs remained stably arrested and preserved all the features of senescent cells despite the addition of estrogen.

## 4. Materials and Methods

### 4.1. EnSC Culture Conditions

The EnSC line used in this study was obtained from the shared research facility “Vertebrate cell culture collection” at the Institute of Cytology of the Russian Academy of Sciences (supported by the Ministry of Science and Higher Education of the Russian Federation, Agreement №075-15-2021-683). Cells were characterized in our previous study [40]. Cells were cultured in DMEM/F12 (Gibco BRL, NY, USA) at 37 °C in a humidified incubator containing 5% CO_2_. The cultural medium was supplemented with 10% FBS (HyClone, Logan, UT, USA), 1% penicillin-streptomycin (Gibco BRL, NY, USA), and 1% glutamax (Gibco BRL, NY, USA). Serial passaging was performed when the cells reached 80–90% confluence. Cells at early passages (5–9) were used in all experiments.

### 4.2. Molecular Cloning

Four-steps molecular cloning was performed to insert the sequence encoding the mCherry fluorescent protein into pLenti CMV/TO RasV12 Puro (w119-1) (#22262, Addgene, Watertown, NY, USA). Firstly, the mCherry sequence, along with the P2A sequence, were amplified from the pUltra-hot lentivector (#24130, Addgene, Watertown, NY, USA) using the specific primers listed in Table 1. Secondly, the HRAS^G12V^ sequence was amplified from the pLenti CMV/TO RasV12 Puro (w119-1) using the specific primers listed in Table 1. Of note, the mCherry reverse primer and the HRAS^G12V^ forward primer contained complementary sites for further overlap. The obtained sequences were then combined by amplification using two primers—mCherry forward and HRAS^G12V^ reverse (annealing temperature 67 °C, 2 min elongation). The final product was inserted into pLenti CMV/TO RasV12 Puro (w119-1) through restriction using XbaI (New England Biolabs, Ipswich, MA, USA) and BamHI (New England Biolabs, Ipswich, MA, USA), followed by ligation using Quick Ligation™ Kit (New England Biolabs, Ipswich, MA, USA). The obtained lentivector was named HRAS-mCherry pLenti CMV/TO RasV12 Puro.

For CRISPR-mediated PTEN knockout, we used pCC_01—hU6-BsmBI-sgRNA(EþF)-barcode-EFS-Cas9-NLS-2A-Puro-WPRE (#139086, Addgene, Watertown, NY, USA). Oligonucleotide sequences of single guide RNAs (sgRNAs) for *PTEN* were designed using the CCTop-CRISPR/Cas9 target online predictor and the CRISPR-ERA web applications; sequences are presented in Table 1. SgRNA coding sequences were inserted into the above-indicated lentivector using BsmBI-based cloning (Thermo Fisher Scientific, Waltham, MA, USA), following the protocols described in our previous study [18]. Unmodified lentivector, containing a non-targeting sgRNA sequence, was used as the non-targeting control.

All amplification procedures were performed using the Encyclo Plus PCR kit (Evrogen, Moscow, Russia). DNA products were purified from PCR mix/agarose gels using the Cleanup Standard kit (Evrogen, Moscow, Russia). The plasmid DNA was extracted using the Plasmid Miniprep kit (Evrogen, Moscow, Russia). All procedures were performed according to the manufacturer’s instructions. Plasmids were amplified using *E.coli* strain Stbl3.

### 4.3. Lentiviral Transduction and Tetracycline Treatment

EnSCs were transduced with the viruses produced using the following lentivectors: (1) pLenti CMV/TO RasV12 Puro (w119-1), (2) FgH1tUTG (#70183, Addgene, Watertown, USA), (3) HRAS-mCherry pLenti CMV/TO RasV12 Puro, (4) pCC_01—hU6-BsmBI-sgRNA(EþF)-barcode-EFS-Cas9-NLS-2A-Puro-WPRE, and (5) its variant for *PTEN* knockout. Protocols for the production of lentiviral particles and EnSC lentiviral transduction are described in detail in our previous article [18]. To induce HRAS^G12V^ expression, EnSCs transduced with the appropriate lentiviruses were cultured in medium containing 20 µM tetracycline (Sigma-Aldrich, St. Louis, MO, USA). Tetracycline-containing medium was changed every two days.

### 4.4. Cells Treatment Conditions

All experimental treatments were performed in complete culture media; either inhibitor was added simultaneously with tetracycline every other day. The following inhibitors were used: 20 µM LY294002 (LY) (Sigma-Aldrich, St. Louis, MO, USA), 10 µM U0126 (Sigma-Aldrich, St. Louis, MO, USA), 5 µM SB203580 (SB) (Sigma-Aldrich, St. Louis, MO, USA), 50 µM pifithrin-α (PFT) (Merck, Darmstadt, Germany). All inhibitors were dissolved in DMSO, following the manufacturer’s instructions. For estrogen treatment, EnSCs were treated with 10 nM β-Estradiol (Sigma-Aldrich, St. Louis, MO, USA).

### 4.5. Flow Cytometry

Measurements of proliferation, cell size, and autofluorescence (lipofuscin accumulation) were carried out by flow cytometry. Flow cytometry was performed using the CytoFLEX (Beckman Coulter, Brea, CA, USA) and the obtained data were analyzed using CytExpert software version 2.0 (Beckman Coulter, Brea, CA, USA). Adherent cells were rinsed twice with PBS and harvested by trypsinization. Detached cells were pooled, resuspended in fresh medium, and stained with 0.1 µg/mL 4′,6-diamidino-2-phenylindole (DAPI, Invitrogen, Carlsbad, CA, USA). DAPI-negative (living) cells were then counted and analyzed for autofluorescence and forward light scattering (reflecting cell size).

### 4.6. SA-β-Gal Staining

SA-β-gal staining was performed using the senescence β-galactosidase staining kit (Cell Signaling, Danvers, MA, USA) according to the manufacturer’s instructions. Quantitative analysis of images was produced with the application of MatLab package. For each experimental point, no less than 50 randomly selected cells were analyzed.

### 4.7. Western Blotting

Western blotting was performed as described previously [15]. SDS-PAGE electrophoresis, transfer to nitrocellulose membrane, and immunoblotting with ECL (Thermo Scientific, Waltham, MA, USA) detection were performed according to the standard manufacturer’s protocols (Bio-Rad Laboratories, Hercules, CA, USA). Antibodies against the following proteins were used: glyceraldehyde-3-phosphate dehydrogenase (GAPDH) (clone 14C10) (Cell Signaling, Danvers, MA, USA), pp53 (Ser15) (clone 16G8) (Cell Signaling, Danvers, MA, USA), p21^WAF/CIP^ (clone 12D1) (Cell Signaling, Danvers, MA, USA), p16^INK4a^ (Affinity Biosciences, Cincinnati, USA), pRb (Ser807/811) (Cell Signaling, Danvers, MA, USA), pChk2 (Thr68) (Cell Signaling, Danvers, MA, USA), pERK (Thr202/Tyr204) (Cell Signaling, Danvers, USA), pAkt (Ser473) (Cell Signaling, Danvers, MA, USA), pp38 MAPK (Thr180/Tyr182) (clone D3F9) (Cell Signaling, Danvers, MA, USA), PTEN (clone 2F4C9) (Invitrogen, Carlsbad, CA, USA), horseradish peroxidase-conjugated goat anti-rabbit IgG (Cell Signaling, Danvers, MA, USA), and horseradish peroxidase-conjugated goat anti-mouse IgG (Cell Signaling, Danvers, MA, USA). Scans of uncropped blots presented in the study are available in Supplementary Figure S1. Each experiment was repeated at least three times, and the representative images are depicted in the figures. For densitometry quantitation, the generated films were scanned and the images were processed using the Scion Image program (v.4.0.2) and the Gelplot2 script. Quantification results are presented in Supplementary Figure S2.

### 4.8. Immunofluorescence

Cells grown on coverslips were fixed with 4% formaldehyde (15 min), permeabilized with 0.1% Triton X-100 (10 min) and blocked with 1% bovine serum albumin (1 h). Cells were incubated with primary anti-γH2AX antibodies (Abcam, Cambridge, USA) overnight at 4 °C, followed by incubation with secondary antibodies—Alexa Fluor 488 goat anti-mouse (Invitrogen, Carlsbad, CA, USA) for 1 h at room temperature. The slides were counterstained with 1 µg/mL DAPI, mounted using 2% propyl gallate, and analyzed using Zeiss LSM Pascal 5 laser scanning microscope (Carl Zeiss, Oberkochen, Germany). ZOE Fluorescent Cell Imager (Bio-Rad Laboratories, Hercules, CA, USA) was used to acquire images of live cells expressing fluorescent reporter proteins.

### 4.9. RNA Extraction, Reverse Transcription, and Real-Time PCR

RNA extraction, reverse transcription, and real-time PCR were performed as described in our previous study [17]. Reagents for RNA extraction (ExtractRNA reagent), for reverse transcription (MMLV RT kit) and for real time PCR (HS SYBR kit) were obtained from Evrogen, Moscow, Russia. Gene expression levels were assessed using the Realtime PCR BioRad CFX-96 amplifier (Bio-Rad Laboratories, Hercules, CA, USA). The following analysis of the obtained data was performed using the Bio-Rad CFX Manager software (Bio-Rad Laboratories, Hercules, CA, USA). Primer sequences and the corresponding annealing temperatures are listed in Table 2.

### 4.10. Statistical Analysis

To obtain significance in the difference between two groups, Welch’s *t*-test or Wilcoxon test were applied. For comparisons among multiple groups, ANOVA or Kruskel–Wallis tests were used. Statistical analysis was performed using GraphPad Prism version 8.0.5.

## 5. Conclusions

To sum up, in the present study, we discovered that senescence is the primary and consistent response of EnSCs to the oncogene expression. The activation of MEK/ERK, PI3K/AKT, p53/p21^WAF/CIP^/Rb, and p38/p16^INK4a^/Rb signaling cascades governed the initiation and development of OIS in EnSCs. The inhibition of either pathway, as well as estrogen supplementation, did not prevent cell cycle arrest in HRAS^G12V^-expressing cells. Finally, we revealed that PTEN loss-induced senescence may serve as a possible reserve molecular mechanism to prevent transformation of HRAS^G12V^-expressing EnSCs. The results obtained suggest that OIS is the defense mechanism underlying the resistance of EnSCs against oncogenic transformation. The limitations of this study include the use of a single EnSCs line and a single model of OIS. Further investigations of the reactions of EnSCs to oncogenic signals should involve replicating the experiments using additional EnSCs lines and different models of OIS. Nevertheless, our findings, at least in part, may explain the rare incidence of endometrial stromal tumors.

Importantly, further precise investigation is required to understand EnSCs senescence in relation to the initiation and progression of endometrial epithelial cancer. A growing body of evidence highlights that senescent stromal cells within the tumor microenvironment are capable of interacting with tumor cells. Though paracrine action, senescent cells might be involved in tumor initiation and progression reviewed in [41]. In line with this speculation, a recent study revealed a significant correlation between stromal p16^INK4a^ expression and endometrial carcinomas, rather than with benign and preneoplastic lesions [42]. The authors concluded that stromal expression of p16^INK4a^ is involved in the development and progression of endometrial carcinoma. Bearing in mind that increased expression of p16^INK4a^ is the most common marker of senescent cells in vivo, it can be logically assumed that senescent EnSCs might have a protumorigenic influence on the epithelial component of the endometrium. However, this assumption remains to be elucidated in future studies.

## Figures and Tables

**Table 1:** Oligonucleotide sequences for mCherry and sgRNAs cloning.

#	Oligonucleotide	Sequence	Tm, °C
1	*mCherry* forward	5′-ATATTTGGATCCGGTCCGATCCACCGGTCGC-3′	63.0
2	*mCherry* reverse	5′-CGTCATCGCTCCAGAAGGCCCGGGATTCTCCTCC-3′	63.0
3	*HRAS^G12V^* forward	5′-GGGCCTTCTGGAGCGATGACGGAATATAAGCTGGTGG-3′	64.0
4	*HRAS^G12V^* reverse	5′-GTCGAGCGGCCGCCACTGTG-3′	64.0
5	*PTEN* sgRNA forward	5′-CACCGAAACAAAAGGAGATATCAAG-3′	-
6	*PTEN* sgRNA reverse	5′-AAACCTTGATATCTCCTTTTGTTTC-3′	-

**Table 2:** Primer oligonucleotide sequences.

#	Oligonucleotide	Sequence	Tm, °C
1	*GAPDH* forward	5′-GAGGTCAATGAAGGGGTCAT-3′	57.0
2	*GAPDH* reverse	5′-AGTCAACGGATTTGGTCGTA-3′	57.0
3	*PTEN* forward	5′-TTGAAGACCATAACCCACCA-3′	58.0
4	*PTEN* reverse	5′-CACATAGCGCCTCTGACTG-3′	58.0
5	*STC1* forward	5′-TGAGGCGGAGCAGAATGACT-3′	59.5
6	*STC1* reverse	5′-CAGGTGGAGTTTTCCAGGCAT-3′	59.5
7	*IL6* forward	5′-ATGTAGCCGCCCCACACA-3′	58.0
8	*IL6* reverse	5′-CCAGTGCCTCTTTGCTGCTT-3′	58.0
9	*MMP2* forward	5′-AGATCTTCTTCTTCAAGGACCGGTT-3′	59.5
10	*MMP2* reverse	5′-GGCTGGTCAGTGGCTTGGGGTA-3′	59.5

## Data Availability

All data generated or analyzed during this study are included in the manuscript.

## Supplementary Materials

The following supporting information can be downloaded at: https://www.mdpi.com/article/10.3390/ijms241814089/s1.

## Abbreviations

EnSCs	human MSCs derived from endometrium
DAPI	4′,6-diamidino-2-phenylindole
DDR	DNA damage response
HSD	honestly significant difference
OIS	oncogene-induced senescence
SASP	senescence-associated secretory phenotype
SA-β-Gal	senescence-associated β-Galactosidase
