# Supplementary material for: Oncogene-Induced Senescence Is a Crucial Antitumor Defense Mechanism of Human Endometrial Stromal Cells
Source: Int J Mol Sci. 2023 Sep 14;24(18):14089. doi: 10.3390/ijms241814089 (PMC10531323; doi:10.3390/ijms241814089)
Supplement: Supplementary file 1 [file ijms-24-14089-s001.zip › ijms-2550067-supplementary.pdf]

International Journal of *Molecular Sciences*

Oncogene-Induced Senescence is a Crucial Antitumor Defense Mechanism of Human Endometrial Stromal Cells

A.L. Toropov [1], P.I. Deryabin [1], A.N. Shatrova [2], A.V. Borodkina [1],*

[1] Mechanisms of Cellular Senescence Group, Institute of Cytology of the Russian Academy of Sciences, Tikhoretsky Ave. 4, 194064 Saint-Petersburg, Russia
[2] Laboratory of Intracellular Membranes Dynamic, Institute of Cytology of the Russian Academy of Sciences, Tikhoretsky Ave. 4, 194064 Saint-Petersburg, Russia
* Correspondence: borodkina618@gmail.com; Tel.: +7-981-680-14-03

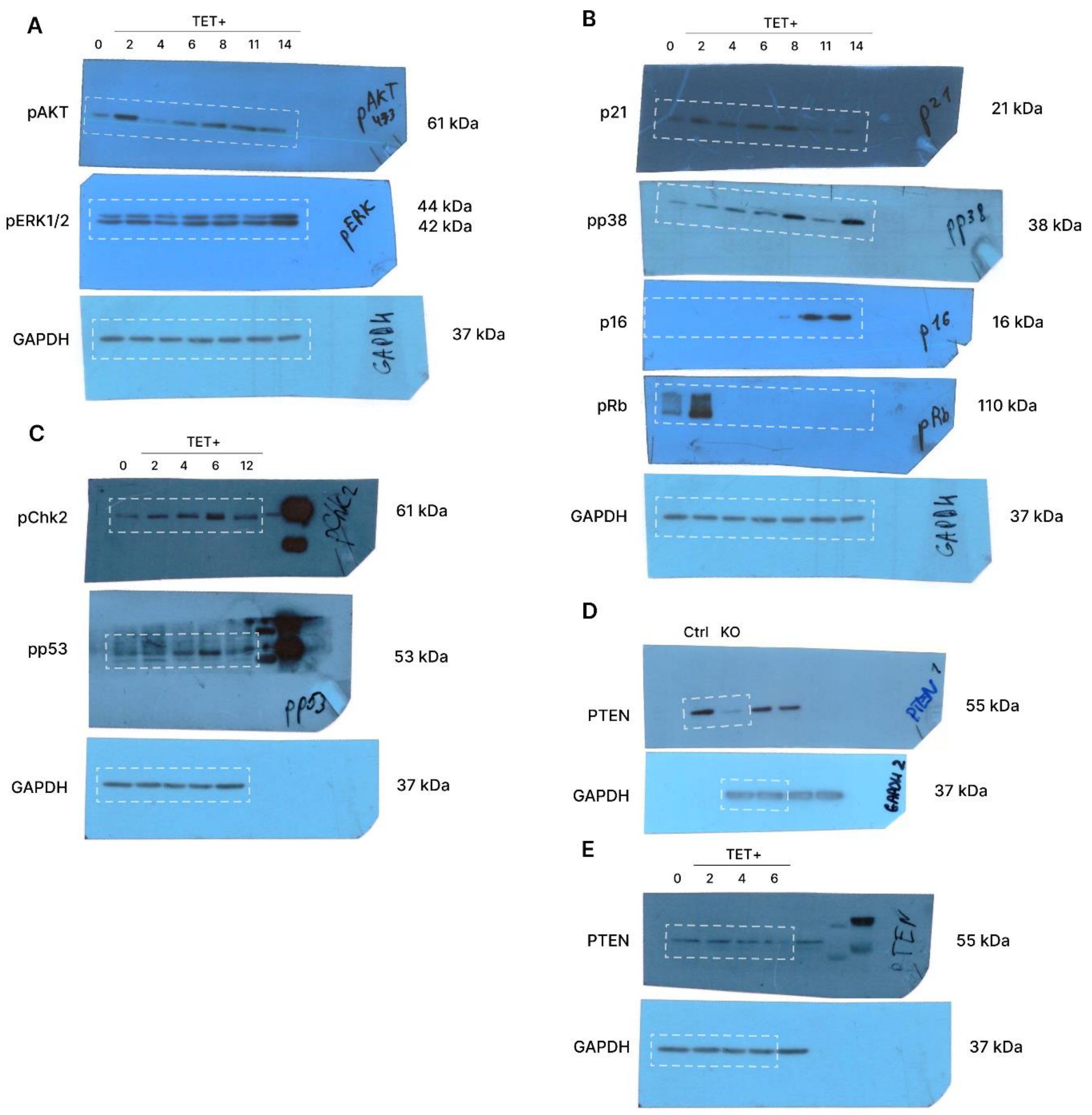

Supplementary Figure S1. Full-length blots presented in the article. (**A**) Blots for Figure 4A, (**B**) Blots for Figure 4D, (**C**) Blots for Figure 4E, (**D**) Blots for Figure 6D, (**E**) Blots for Figure 6B.

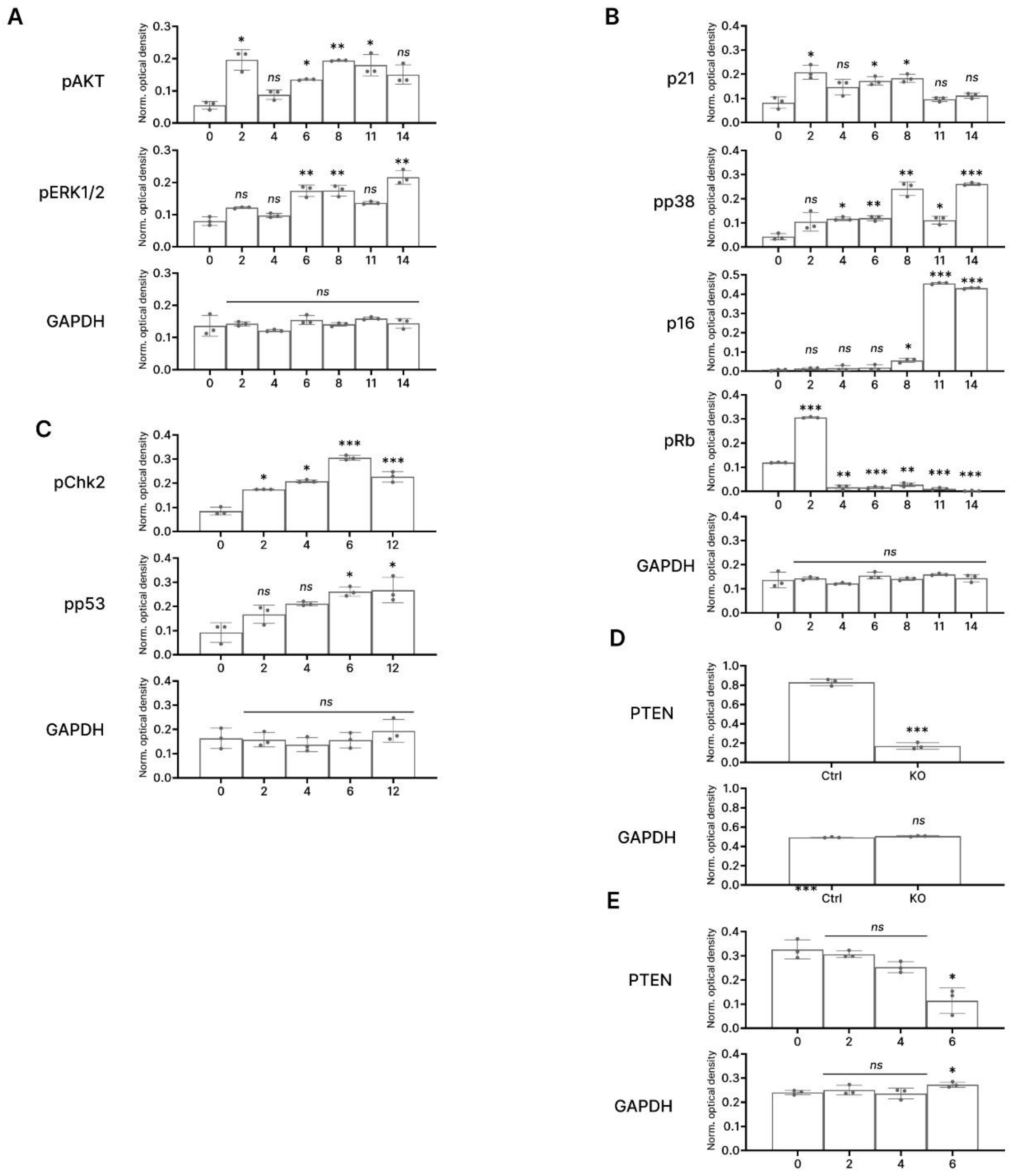

Supplementary Figure S2. Densitometry quantitation for Western blot analysis performed in the study. Estimated optical densities of bands were normalized by sum, each protein / protein modification were analyzed in 3 replicates. (**A**,**B**,**C**) Quantitation results for blots presented at Figure 4. (**D**,**E**) Quantitation results for blots presented at Figure 6. Data are presented as mean ± SD. ns $p \geq 0.05$, * $p < 0.05$, ** $p < 0.01$, *** $p < 0.005$ using Welch's t-test.
